# Experiences of violence among people with severe mental illness: social, biographical and institutional factors (EVIO). A study protocol for a mixed-methods study

**DOI:** 10.3389/fpsyt.2026.1786850

**Published:** 2026-04-22

**Authors:** Silvia Krumm, Anita Scheuermann, Claudia Helmert, Paula Kittelmann, Melanie Pouwels, Nicolai Hojka, Jana Karp, Georg Schomerus

**Affiliations:** 1 Department of Psychiatry and Psychotherapy II, Ulm University, Ulm, Germany; 2 Department of Psychiatry and Psychotherapy, University of Leipzig, Leipzig, Germany; 3 “Vorurteilsfrei” registered non-profit association, Leipzig, in cooperation with the Universities of Ulm and Leipzig, Leipzig, Germany

**Keywords:** mental health professionals, mental health service users, mental illness, mixed-methods study, prevalence rate, qualitative study, victimization, violence

## Abstract

**Background:**

People with severe mental illness (SMI) face a significantly higher risk of victimization than the general population. Beyond physical harm, such experiences can severely impact mental health and recovery processes. Empirical research on violence prevalence among people with SMI in Germany is limited, with little understanding of mechanisms, the impact of gender, and the victim-perpetrator overlap. In order to develop successful prevention strategies against victimization, a comprehensive understanding of the complex phenomenon of victimization is needed. The study aims to develop a comprehensive understanding of the biographical, socio-cultural and institutional context factors related to experiences of violence, as well as to identify barriers to addressing such experiences within mental health settings.

**Methods:**

In a mixed-method study incorporating a quantitative survey and qualitative instruments, we investigate the prevalence of violence, including both victimization and perpetration, among people with SMI. Our study includes the following: (1) In a sample (n=500) of people with SMI, treated in inpatient and outpatient mental health settings, we will investigate the prevalence of victimization and violent behavior, (self-)stigmatization, and barriers for disclosure of experiences of violence within mental health settings. Data will be obtained from samples at seven psychiatric hospitals with mandatory care on a fixed day. For comparisons with the general population, we use an online sample (n=1000). (2) Based on this, we conduct qualitative interviews with a subsample of people with SMI (n=30) who reported experiences of violence. Using biographic-narrative interviews, we focus on the biographical and socio-cultural context (3). In addition, we use qualitative and quantitative measures to investigate mental health professionals’ experiences, institutional factors, and strategies to deal with mental health service users’ violence experiences.

**Discussion:**

Developing interventions to prevent and reduce victimization requires a systematic assessment of mental health service users’ violence experiences and a comprehensive understanding of the biographical, socio-cultural, and institutional factors. Our study aims to contribute fundamentally to a deeper understanding of violence experiences in Germany, thereby providing the basis for the development of targeted interventions to improve the awareness and the handling of violence experiences among a specific vulnerable group.

**Trial registration:**

This study is registered in the German Clinical Trial Register (DRKS) and the WHO International Clinical Trials Registry Platform (ICTRP) under registration number DRKS00032041 (Date of registration July 2023).

## Introduction

Compared to general population data, people with severe mental illness (SMI) are more likely to experience physical and/or sexual violence (1, 2). A meta-analysis indicated a 5-fold relative odds for any physical violence in people with SMI compared to those without SMI (2). Regarding the prevalence and risk of sexual violence, a recent meta-analysis revealed high pooled prevalence estimates and increased odds in male and female psychiatric service users compared to non-psychiatric service users (1). Violence experiences cause not only physical harm but can also have detrimental effects on mental health, predisposing individuals to the onset and severity of psychosis (3, 4) and leading to self-stigmatization (5). Experiences of violence are often shaped by biographical trajectories, unfolding across the life course. While early victimization increases the risk of developing mental health problems later in life, the likelihood of further victimization rises after the onset of mental illness. A Danish cohort study, including more than 2 million individuals, observed an increase in the incidence of victimization following the onset of mental illness (6). Studies on victimization among people with SMI have identified a number of risk factors with mixed findings on the role of psychopathology, cognitive deficits, diagnoses and substance (mis)use (7–10). Social risk factors include unemployment and homelessness (9), poverty (11) and social exclusion (12).

Regarding gender, evidence suggests that women and men with SMI experience similarly high rates of physical violence (2). This is noteworthy, as population data typically show higher prevalences of physical victimization among men (13), indicating that the gender difference observed in the general population is reduced among people with SMI (2). For sexual violence, while women with SMI experience higher rates of victimization than men with SMI, the gender gap is narrower than in the general population (2). Overall, both women and men with SMI face markedly elevated risks of violence compared to the general population (2).

Relative incidence rates confirm women’s particular vulnerability to violence: With the onset of a mental illness, women’s risk of experiencing violence increases by 2.7 times (compared to a 1.8 times enhanced risk in men) (6). Women with SMI also face a significantly heightened risk of sexual violence: Compared to women in the general population, they are nearly ten times more likely to be victims of sexual violence (2). Studies which systematically address the role of gender for experiences of violence among individuals with SMI are still rare.

Sociocultural factors play a crucial role in shaping the vulnerability and experiences of individuals with SMI in the context of violence. Ethnicity and migration background are relevant when investigating violence experiences among individuals with SMI. Refugees and asylum seekers have been found to frequently experience violence and related health problems, with elevated rates of both physical and sexual victimization as well as mental health conditions (14). Thus, racial and ethnical marginalization needs to be considered as a potential risk factor for (re)victimization (15). In addition, prevailing societal and professional perceptions of mental illness may also play a role in violence experiences among this group. The psychiatric discourse on violence is often characterized by notions of higher prevalence of perpetration than victimization and by notions of a perpetrator-victim dichotomy, implying that individuals are either perpetrators or victims. However, a recent systematic review on interpersonal violence perpetration and victimization risk within single study cohorts provided evidence for higher rates of victimization than perpetration among both individuals with SMI and those in the general population, and for higher rates of both victimization and perpetration among individuals with SMI compared to the general population (16). Empirical studies indicate an overlap between victimization and perpetration. A secondary analysis of five U.S. studies (total sample n=4474) revealed that, compared to individuals who only experience violence (13.3%) or only perpetrate violence (6.3%), those who both experienced and perpetrated violence (17.6%) were more prevalent (17). This aligns with the results of a Swedish registry study, which found a significantly higher risk of combined victimization and perpetration among individuals with a psychiatric diagnosis compared to isolated victim or perpetrator experiences (7). According to a meta-analysis of studies on violence and mental illness, previous experiences of violence increased the risk of perpetration by a factor of 6 (18). A prospective study demonstrated that experiences of violence within the past six months significantly increased the risk of perpetrating violence in the following year, outweighing other risk factors (19). To date, beyond prevalence rates, little is known about the forms and mechanisms of overlapping perpetrator-victim perspectives among individuals with SMI.

Mental health professionals are in a central position to identify victimization among service users and to provide adequate support. However, despite the strong evidence for the increased risk among people with SMI and the detrimental effects of victimization for individuals and their families (20), victimization among people with SMI is often neither systematically recorded nor considered in mental health care (21–23). Victims of violence identify key barriers to disclosure, including feelings of shame and guilt, delayed realization of the violence, fear of subsequent violence, and inappropriate reactions to the disclosure (24). Mental health professionals report feeling overwhelmed and uncertain in recognizing and dealing with experiences of violence, fearing negative consequences for the victims, or refer to practical barriers such as a lack of time (24). In an Australian study, professionals were found to have varying responses to disclosures of sexual assaults, including disparaging views that led to minimizing, ignoring, or blaming the disclosures, and while some acknowledged the prevalence of sexual violence, they felt ill-equipped to respond appropriately (25). The lack of mental health professionals’ engagement with the phenomenon of service users’ victimization brings about three significant problems: First, it impedes the early detection and addressing of experiences of violence in medical history taking and therapy, which is crucial for trauma-therapeutic interventions and support, e.g. connecting to specialized services (26). Second, there is a risk for re-traumatization that can occur in inpatient settings, particularly in regard with coercive measures. Seclusion and/or restraint can have deleterious physical and psychological consequences including post-traumatic stress disorders particularly for people with previous experiences of victimization (26). However, studies on re-traumatizing experiences during inpatient treatment are scarce, and the role of gender remains widely unclear. Third, the lack of awareness and engagement of mental health service providers hinders the development of structural measures to prevent and reduce the risk of people with SMI becoming victims of violence within and without mental health settings. As the outlined background on victimization of people with SMI demonstrates, developing effective measures to prevent victimization requires a comprehensive understanding of the complex and multifaceted underlying mechanisms of violence, as well as an understanding of the factors influencing the disclosure of such experiences in mental health settings.

According to Thom et al. (27) around 37.9% of the population are affected by a mental illness. Recent reviews emphasize that despite evidence of elevated victimization rates among people with SMI, representative prevalence studies that allow for cross-country comparisons and population-based estimates in Germany remain scarce (28). Moreover, more research is needed to provide a comprehensive picture of the risk variables influencing victimization experiences (29). Taken together, these gaps highlight the need for further research that combines representative prevalence data with an analysis of social, biographical and institutional factors to provide a more comprehensive understanding of victimization among people with SMI.

## Methods

### Study aim

The study aims to provide a comprehensive analysis of violence experiences including victimization and perpetration amongst people with SMI including:

1. Frequencies, forms, contexts, and disclosure of physical and sexual violence experiences among people with SMI in Germany, including overlaps between victimization and perpetration.
2. Impact of gender, biographical and socio-cultural factors correlations; and the subjective meanings and attributes of violence experiences.
3. Mental health professionals’ views on violence experiences among mental health service users.

### Epistemological and methodological framework of the study

This mixed-method study is positioned at the intersection of sociological violence research, social psychiatry, and gender studies. Social psychiatry emphasizes the social, biographical, and cultural influences on the development, course, and management of mental illness, and highlights the reciprocal relationships between subjects and their environments (30). In turn, sociological violence research conceptualizes violence as an intentional act of power embedded in social orders and shaped by situational and contextual conditions (31). Following Popitz’s definition of violence as an intentional act leading to physical harm (32), the study focuses on physical violence (e.g., hitting, kicking, robbery, armed assault) and sexual violence (e.g., harassment, attempted rape), including domestic and partner violence. Methodologically, the study combines context-centered approaches, which examine macro-social and cultural conditions (e.g., poverty, norms, inequality), with situation-centered approaches, which reconstruct interactions and subjective interpretations of violence on the micro-sociological level. Particular emphasis is placed on the biographical embedding of violent experiences, recognizing their temporal structuring and dynamic unfolding across the life course. By linking social, institutional, and biographical factors, this design allows us to explore how prior experiences, life trajectories, and environmental contexts intersect to shape patterns of victimization among people with severe mental illness. From a gender perspective, the framework draws on feminist and sociological theories that interpret male violence against women as an expression of dominance, while situating violence among men within the construction and negotiation of hegemonic masculinities (33). This dual lens enables the analysis of both hetero- and homosocial dimensions of violence. At the same time, the framework transcends dichotomous attributions of (male) perpetrators and (female) victims by addressing victim-offender overlaps and the situational as well as biographical dynamics that link victimization and violent behavior. In this way, mechanisms of inversion between victim and perpetrator roles can be identified, and the impact of socialization processes or continuous exposure to violence on trajectories of vulnerability and violent behavior can be systematically addressed.

### Study design

We conduct a cross-sectional mixed-method study consisting of three subprojects. The mixed-method-design will be realized by combining quantitative survey data (subproject 1) and subsequent qualitative narrative-biographical interviews (subproject 2) in line with an Explanatory Sequential Design (34). Qualitative focus groups, interviews and subsequent survey data (subproject 3) will be combined according to the Exploratory Sequential Design (34).

- Subproject 1: Quantitative cross-sectional survey with mental health service users.
- We will conduct a quantitative cross-sectional study involving 500 people with SMI through a key date survey conducted in various psychiatric clinics. Additionally, a representative sample of 1000 individuals from the general population will be surveyed by USUMA, a German market and social research institute.
- Subproject 2: Qualitative study with mental health service users.

We will conduct a qualitative study using narrative-biographical interviews data involving a subsample of approximately 30–40 people with SMI from subproject 1.

- Subproject 3: Qualitative study and quantitative survey with mental health professionals.

In a first step, we will collect qualitative data through approximately 30 semi-structured interviews and four focus groups with mental health professionals. In a second step, we collect quantitative data from approximately 100 mental health professionals.

### Study setting

Study participants will be recruited at the following study sites providing inpatient and outpatient mental health treatment and care in Germany:

- Department of Psychiatry and Psychotherapy II, Ulm University, District Hospital Guenzburg, Germany.
- Psychosomatic Medicine and Psychotherapy, Ulm University, Germany.
- Department of Psychiatry, Psychotherapy and Psychosomatics, Augsburg University, District Hospital Augsburg, Germany.
- Department of Psychiatry and Psychotherapy, Leipzig University, Germany.
- Department of General Psychiatry and Psychotherapy, Center for Psychosocial Medicine, The Specialist Hospital Bethanien Hochweitzschen, Germany.
- Department of Psychiatry and Psychotherapy, Helios Hanseklinikum Stralsund, Germany.
- Department of Psychiatry, Psychosomatics and Psychotherapy, Helios Park Hospital Leipzig, Germany. Clinic for Psychiatry, Psychotherapy and Psychosomatics, Saxon Hospital Altscherbitz, Germany.

### Participants and eligibility criteria

The standardized survey of subproject 1 includes people with SMI of legal age (18+ in Germany) who are undergoing treatment in one of the participating clinics at the time of recruitment. The term “people with SMI” refers to individuals diagnosed with mental illness that significantly impair their ability to function in daily life. These conditions often include disorders such as schizophrenia, bipolar disorder, or severe major depression, and typically require (long-term) treatment and support (35). For our study, we operationalize the term “people with SMI” based on the indication for psychiatric hospitalization, as well as outpatient treatment in a psychiatric hospital or outpatient service, which is considered an indicator of “severe mental illness”. Synonymously we use the term “mental health service user”. Exclusion criteria include language barriers and cognitive deficits, such as dementia or intellectual disabilities.

The qualitative study of subproject 2 includes persons of legal age who took part in the standardized survey in subproject 1. The selection of participants is based on the extent of violence experienced as indicated in the standardized questionnaire. Participants who report experiences of physical or sexual violence will be considered eligible for the qualitative interviews. The phrase “beyond a cut-off” refers to the self-reported occurrence of at least one experience of physical or sexual violence in the questionnaire. Final selection will additionally consider the forms of violence reported and sociodemographic characteristics in order to ensure heterogeneity of cases.

Individuals who meet these criteria and agree to participate in a narrative-biographical interview will be included. In subproject 3, mental health professionals from the fields of nursing, psychology, social work, and medicine who are employed at one of the participating clinics will be recruited for the qualitative interviews and focus groups, and for the subsequent quantitative survey.

### Participant timeline

The recruitment period for participants in subproject 1 has started in January 2024 and will continue until August 2025. At the time of submitting this protocol, 250 participants have been included in the study. The recruitment of participants for the narrative-biographical interviews in subproject 2 has started in August 2024, with the interviews set to be conducted until August 2025. Participants for the qualitative interviews in subproject 3 have been recruited since March 2024, with the recruitment period scheduled to end in February 2025. At the time of the submission of this protocol, (n=29) interviews have been conducted. Participants for the focus groups in subproject 3 are scheduled to begin in December 2024 and will be conducted until June 2025. Participants for the questionnaire survey in subproject 3 will be included in the study from October to December 2025.

### Outcomes

#### Subproject 1 (quantitative cross-sectional survey with mental health service users)

The primary outcome of subproject 1 is the frequency of violence experiences among people with SMI, both as victims and perpetrators, compared to a control group from the general population. The study examines correlates and contextual factors of violence, such as gender, socioeconomic status, social support, coping resources, and psychopathology. The secondary outcomes include the impact of internalized stigmatizing beliefs among people with SMI, especially in relation to violence experiences. Furthermore, barriers of disclosure will be assessed, focusing in whom these experiences have been communicated to, why or why not, and whether they have been incorporated into treatment if reported in a clinical setting.

#### Subproject 2 (qualitative study with mental health service users)

The aim of subproject 2 is to gain a deeper understanding of the subjective meaning of violence experiences from a biographical perspective with a focus on gender and victim-perpetrator-dynamics. We are interested in both the processual nature of violence within the biographical context and the biographical processes as a result of violence experiences. Particular attention will be paid to biographical continuities, breaks, and changes in the narratives of violence experiences, including the phenomenon of serial victimization and re-victimization both within and outside of mental health services. Furthermore, we will pay particular attention to normative expectations, perceptions of stigma in relation to violence experiences, and the interrelation between stigmatization and the disclosure of violence experiences.

#### Subproject 3 (qualitative study and quantitative survey with mental health professionals)

The aim of subproject 3 is to analyze the experiences and attitudes of mental health professionals regarding violence experiences among mental health service users and to compare these views with those of the service users themselves concerning their experiences of violence. We build on existing findings related to the barriers and facilitating factors identified by professionals concerning the disclosure of experiences of violence. We are particularly interested in the individual (experiences, attitudes) and contextual factors (setting) from the professionals’ perspective that can influence disclosure within the context of psychiatric treatment, with a specific focus on the role of gender in dealing with the service users’ experiences of violence.

### Recruitment and sample size calculation

#### Subproject 1 (quantitative cross-sectional survey with mental health service users)

Focusing on people in psychiatric treatment allows us to survey a representative sample of a defined population. This group represents those with immediate access to therapeutic interventions, and therefore to potentially address experiences of violence. A sufficiently large sample is targeted to enable adequate analyses of different context factors. The sample size of n=500 is chosen to enable the most precise estimation possible of the prevalence of violence experiences.

For statistical power analysis regarding the sample size in subproject one, considering the overall exploratory nature of this study, the variety and theoretical parity of the variables of interest (no primary predictor), and the scarcity of published data that could guide a strict *a priori* power analysis, we liberally set the sample size to 500 or 100 per clinic, respectively. This sample size is sufficient to detect group differences of medium to large effect sizes with a maximum error rate of 5% for both Type I (alpha) and Type II (beta) errors. We illustrate this with the analysis of clinic-level effects using G*Power 3.1.9.7 (36). Based on 500 data points, global parametric comparisons for independent groups can detect site effects on metric variables such as respondent’s age down to f=0.20 with a power of at least 95% (one-way ANOVA with fixed effects, 5 equally-sized groups). This corresponds to site effects on ordinal (e.g. socioeconomic status), nominal (e.g. marital status), or binary variables (e.g. gender) of w≥0.20 (Chi^2^-distribution based tests, df=4). For comparison, a previous study by Johnson et al. (2015) (37) found only a marginal effect of gender on participant status as victim, perpetrator or victim/perpetrator compared to people who did not experience violence (w=0.06, p<0.01). However, comparability of this study’s sample with our target population is strongly limited, as men and people with schizophrenia were overrepresented (59.8% and 63.4%, respectively). For our study, we expect considerably more women than men in psychotherapeutic services (38) and lower rates of schizophrenia, given a prevalence rate of less than 1% in Germany (27).

Participants will be recruited at the following cooperating hospitals: Clinic and Polyclinic for Psychiatry and Psychotherapy, University Hospital of Leipzig (urban area) (n=100); “Helios Park” - Hospital Leipzig (urban area) (n=100); District Hospital Guenzburg (small town/rural area) (n=100); Specialized Hospital Bethanien Hochweitzschen (rural area) (n=100); Helios Hanseatic Hospital Stralsund (rural area) (n=100). Since violence is a taboo topic, we expect that many people may not want to participate in our study. In order to still obtain a sufficiently large sample size to calculate the aforementioned statistical parameters, we have additionally collaborated with the following two clinics: The Clinic for Psychosomatic Medicine and Psychotherapy at the University Hospital Ulm (urban area). Further, we cooperate with the Clinic for Psychiatry, Psychotherapy and Psychosomatics, Saxon Hospital Altscherbitz, Germany (outskirt of Leipzig). A cooperation has been requested with the Clinic for Psychiatry and Psychosomatics at the District Hospital and the University of Augsburg (urban area).

On a specified key date, the project staff will approach all patients who are receiving partial or full inpatient treatment. For practical reasons, different key dates will be chosen for various treatment units (wards, outpatient clinics). Recruitment will continue in each clinic until the target sample size is reached. Inclusion criteria were legal age (18 years) and German language skills. Exclusion criteria were dementia, intellectual impairment, current psychotic symptoms, and if the person was in isolation or fixation. All patients that meet the inclusion criteria and don’t need to be excluded for above mentioned reasons will be asked if they would agree to fill out the questionnaire. They will be handed the participants information and fill out informed consent (see section “Ethics”).

Furthermore, we will recruit a general population sample (n=1000) through an online survey conducted by USUMA.

The rationale for including a comparison group from the general population is to contextualize prevalence estimates observed among people with SMI and to determine whether observed rates exceed background population levels. The general population sample is recruited via an online survey conducted by USUMA, which enables access to a large and heterogeneous sample and allows quota sampling based on key sociodemographic characteristics (e.g., age, gender, and region), thereby supporting structural comparability between groups.

People with SMI are recruited in psychiatric services and complete the same standardized self-report measures on-site (paper-pencil or tablet-based). Although both samples rely on identical instruments, differences in recruitment context and survey mode (online vs. clinical setting) may introduce potential mode effects, which will be considered as a potential limitation when interpreting group differences in prevalence.

#### Subproject 2 (qualitative study with mental health service users)

Following an explanatory sequential design, the selection of respondents in subproject 2 is guided by the results of the quantitative survey conducted in subproject 1. We will select participants from the subproject 1 sample based on the extent and forms of violence experiences as indicated in the standardized questionnaire, as well as sociodemographic characteristics. The sample size is determined by theoretical saturation of the phenomenon being studied. Based on previous experiences, we anticipate a sample size of 30–40 interviews (n=15–20 women; n=15–20 men).

#### Subproject 3 (qualitative study and quantitative survey with mental health professionals)

To capture both the subjective and collective perspectives of mental health professionals, subproject 3 employs an exploratory sequential design, beginning with qualitative interviews followed by standardized measures. Recruitment takes place at the participating clinics, where staff members from relevant professional groups involved in direct patient care will be invited to take part. For the qualitative study part, we will recruit participants who exhibit a broad range of sociodemographic and professional characteristics (gender, age, professional experience, treatment setting). We anticipate conducting approximately 30 to 40 interviews and about 3 to 4 focus groups. The final sample size will be determined by theoretical saturation. For the following quantitative part, a standardized questionnaire will be administered to assess frequencies and correlations of mental health professionals’ attitudes. Staff members will be invited to participate in an online survey.

### Data collection and study instruments

#### Subproject 1 (quantitative cross-sectional survey with mental health service users)

Study participants complete the questionnaires on a tablet or using paper-pencil in the clinic. Study staff are available for inquiries. Following a participatory approach, the questionnaire was tested and reviewed in collaboration with peer researchers. Data are collected and managed using REDCap electronic data capture tools hosted at Leipzig University. REDCap (Research Electronic Data Capture) is a secure, web-based software platform designed to support data capture for research studies, providing 1) an intuitive interface for validated data capture; 2) audit trails for tracking data manipulation and export procedures; 3) automated export procedures for seamless data downloads to common statistical packages; and 4) procedures for data integration and interoperability with external sources (39). We will use the following instruments:

- The Patient Health Questionnaire (PHQ-D) (40) to assess impairments due to physical and psychological symptoms.
- Psychiatric and psychotherapeutic hospitalization and treatment experiences as well as diagnoses and symptoms are queried.
- Substance use is assessed using short forms of AUDIT (41) and DUDIT (42).
- The German version of the Self-Stigma of Mental Illness Scale (SSMI-SF) (43, 44).
- One item to assess the general satisfaction with life (45).
- ISEL-12 (46) to measure social support, and additional items designed with participatory researchers to assess other resources.
- Questions on sociodemographic characteristics: age, nationality, socioeconomic status (47, 48), sex and gender (similar to 49), family status, sexual orientation (similar to 50, 51), and experienced discrimination.
- Social desirability is assessed using the Social Desirability Gamma Scale (52).
- Experiences of violence: a survey on the type, frequency, and context of violence (violence as well as victimization experiences during the last year and over the lifetime), compiled in accordance with (53–55).

Violence was operationalized as experiences of physical violence, sexual violence, and threats of violence in line with definitions from the WHO World Report on Violence and Health (56), and assessed using standardized self-report items adapted from the MacArthur Community Violence Screening Instrument (MCVSI) (53–55, 57). The questionnaire includes items assessing experiences of physical violence (e.g., being hit, kicked, robbed, or threatened with a weapon) and sexual violence (e.g., sexual coercion), based on and adapted from items provided by Harris et al. (54) and components of the MCVSI. Respondents are asked about both lifetime and past-year experiences, as well as the context and relationship to the perpetrator.

#### Subproject 2 (qualitative study with mental health service users)

Selected participants of subproject 1 who consented to be interviewed in the qualitative study part will be contacted and invited to participate. Narrative-biographical interviews will be conducted either face-to-face or via video call, using an encrypted provider that complies with the requirements of the German General Data Protection Regulation (DSGVO). The interviews will be conducted by researchers trained and experienced in qualitative interviewing.

Narrative-biographical interviews enable a valid exploration of individual structures of meaning and significance. The open narrative prompt, focusing on experiences of violence, elicits an autobiographical narration that aligns with the storyteller’s relevance systems not constrained by the researchers’ preconceptions. An interview guide is developed, based on relevant literature and validated through participative qualitative research workshops, which include researchers from diverse mental health fields as well as mental health service users. The use of this guide ensures that relevant aspects are addressed based on the current state of research and (preliminary) findings from subproject 1, e.g. dealing with client’s disclosure of violence experiences. The interviews will be audio-recorded and fully transcribed. Additionally, spontaneous field notes will be taken immediately after the interview as part of the research diary.

#### Subproject 3 (qualitative study and quantitative survey with mental health professionals)

We collect data on the subjective and collective perspectives of mental health professionals on their client’s violence experiences using qualitative interviews, focus groups, and standardized questionnaires.

Qualitative approach: The guidelines for the interviews and focus groups consist of predominantly open-ended questions about everyday experiences, as well as semi-structured questions to enhance comparability. Data analysis is conducted using content-analytical approaches and supplemented with reconstructive analytical tools. The results are validated through intersubjective exchange and consensus alignment of interpretations during regular participatory research workshops.

Quantitative approach: Based on both the findings from the quantitative survey in subproject 1 and initial qualitative findings in subproject 2 and 3, a questionnaire will be developed to assess and quantify professionals’ perspectives on facilitators and barriers related to dealing with their clients’ experiences of violence. Stigmatizing attitudes will be captured by the German Version of the Community-Attitudes-Toward-the-Mentally-Ill inventory (CAMI) (58).

### Promotion of study participation

Potential study participants will be informed about the study in a separate meeting prior to participation, where they will be encouraged to take part. Study staff will introduce the study and explain the procedures face-to-face in either group or individual settings on each ward. Study information and consent forms will be obtained from participants. In addition to the written consent forms, information will be provided in a concise format through a study flyer. Study participants will complete the questionnaires independently, either on a tablet or on paper, while study staff remain nearby to provide support in case of questions or emotional stress reactions. In subproject 1, after completing the survey, participants can choose whether they would like to be re-contacted to participate in subproject 2. If they agree, they will receive an email a few weeks later inviting them to take part in the qualitative study (subproject 2). In the qualitative study parts (subproject 2, subproject 3), participants receive an incentive of 30€ for participation. Participants from the general population will be contacted by USUMA and conclude the survey online.

### Data management

Data entry into the RedCap database will be performed using any device with internet access, allowing for flexible and efficient data collection. To ensure the reliability of the dataset, all entered data will be checked for completeness on a regular basis. The collected data will be accessible to the study team in a pseudonymized and encoded format, maintaining confidentiality and privacy while allowing for effective data analysis. The data manager will back up the dataset at regular intervals, saving it as a CSV file to prevent data loss and ensure the security of the information. Additionally, plausibility checks will be performed to verify the consistency and accuracy of the data. New variables, such as sum scores, will be generated using the statistical software Stata Version 17.0 (59).

The qualitative interviews will be audio-recorded and transcribed for analysis. For transcription, f4x, an online service that complies with the General Data Protection Regulation in Germany (DSGVO) and meets the requirements for medical data, will be used. After transcription, the research staff will review and pseudonymize the transcripts to ensure the confidentiality and the study participants’ personal rights.

### Data analysis

#### Statistical methods

For subproject 1 and subproject 3, we will describe the type, frequency and context of experienced and exerted violence and compare groups by t-test and ANOVAs. Associations will be analyzed by regression and correlation models (60). Further, we consider multilevel models to analyze group-specific association of varying variables with experienced and exerted violence (61, 62). To assess the psychometric characteristics of new developed and translated questionnaires, descriptive analyses and factor analysis will be conducted (60). Finally, we will interpret the qualitative and quantitative data in conjunction and present the results integratively using joint displays (34, 63).

#### Qualitative data analysis

The qualitative data collected in subproject 2 will be analyzed using a hermeneutic-reconstructive methodology to gain deeper insights into the subjective meanings and social contexts within the participants’ narratives. This follows the integrative basic procedure (“Integratives Basisverfahren”) proposed by Kruse (64) and the biographical-analytical approach of Lucius-Höhne and Deppermann (65). Particular attention will be given to the subjective and latent meanings, and the biographical embedding of violence experiences. In addition to the content aspect (factual framework), the sequential text analysis (word-by-word and line-by-line) is carried out at the level of interaction between the interviewer and the interviewee (a), semantics (b), syntax (c) and narrative figures (d), whereby a variety of linguistic-communicative methods, positioning analysis and agency analysis will be applied (64, 65). We aim at the development of case structure hypotheses (“Fallstrukturhypothesen”) and a typology regarding the biographical coping of violence experiences with a special focus on gender and perpetrator-victim dynamics. In line with this, our interpretive analysis will remain attentive to emergent sociocultural themes in participants’ narratives (e.g., migration background, discrimination, family support, religiosity/spirituality) when reconstructing case structures and typologies (66).

This methodological approach is particularly suited to reconstructing case structures in biographical narratives and to capturing the dynamics of victim-perpetrator inversions. To ensure the intersubjective exchange and consensual analysis required for reconstructive analysis, qualitative research workshops will be organized, involving research staff from diverse disciplines and with varying levels of mental health service use experiences.

The qualitative data collected in subproject 3 will be analyzed using qualitative structured content analysis according to Kuckartz (67) and reconstructive analysis according to Kruse (64). For qualitative data management (68) analysis software MAXQDA will be used. In the first step of the content analysis, the transcripts of the first interviews (10–25%) will be coded deductively based on the interview guide topics. Iteratively, the deductive codes and categories will be extended, refined or modified by means of inductive coding. In the second step, the preliminary coding tree will be applied to the entire material and, if necessary, further extended, refined or modified until a final category system is reached.

### Ethics

#### Informed consents

Each potential study participant will be informed verbally and in writing about the study’s aims, procedures, data policies, and potential risks. A copy of the information sheet will be provided to the study participants. After ensuring that they fully understand the study details and all their questions have been answered, informed consent will be obtained separately for each subproject. In subproject 1, participants from the general population will provide informed consent by checking a box at the beginning of the online questionnaire, while patients in psychiatric hospitals will sign an informed consent form. In subproject 2 and subproject 3, participants will send their signed consent form by email or post. Respondents will be assured that they can refuse to participate or withdraw consent at any time without facing any adverse consequences.

#### Confidentiality

Signed consent forms will be kept in a locked file cabinet. Password-protected cloud files will ensure the exchange of information between subprojects. Only collaborating researchers will have access to the stored personal data. Data for analyses will be kept with a cipher and without any personal data. The cipher enables researchers to delete data securely in case of withdraw of consent. The study results will be published anonymously, without the possibility of drawing conclusions about individual participants. Archived data will be archived on servers at the Department of Psychiatry and Psychotherapy at Leipzig University in Germany, and at the Department of Psychiatry and Psychotherapy II at Ulm University in Germany.

#### Harms

As this is an observational study without an intervention, we do not expect adverse events. The study team is prepared and trained to provide tools to help study participants in case of emotional reactions, like dissociation, to remembering challenging life events like experienced and exerted violence. The evaluation team will be trained and provided with structured instructions to recognize and deal with suspicions or statements of suicide of the participants. Researchers will be in regular exchange of information; any reported adverse event will be documented and discussed. In the unlikely case of an adverse event, it will be documented in an encrypted file.

#### Auditing

No external auditing will be provided. The study progress will be discussed in weekly meetings of the research team. Materials including interview guides and questionnaires will be discussed with experienced experts and in research workshops.

#### Protocol amendments

Important changes to the protocol will be submitted to the responsible ethics committee. Changes are updated in the German Clinical Trials Register (69).

## Discussion

Globally, victimization is recognized as one of the pressing problems in the treatment and care of people with SMI (70). Victimization has serious consequences for mental health, leading to poor clinical outcomes and hindering recovery in an already vulnerable group (70–75). To date, effective measures to prevent and reduce victimization among this group are widely lacking. This is in stark contrast to the right to protection from all forms of exploitation, violence and abuse, including gender-based violence, as well as the corresponding obligation to take appropriate measures, as declared in Article 16 of the UN Convention on the Rights of Persons with Disabilities (76). The low awareness and lack of interventions against victimization in mental health treatment and care further contradict the core principles of social psychiatry, which emphasize the significant role of social and biographical factors in the treatment and care of mental health service users. The development of successful preventive measures requires a comprehensive understanding of victimization. With our mixed-methods study, we aim to address several research gaps:

1. Although internationally established findings exist regarding the increased risk of victimization among people with SMI and the severe consequences for mental health and recovery processes, there is a scarcity of empirical data in Germany concerning the prevalences and forms of physical or sexual violence experiences among this group. The transferability of findings from international studies is limited due to differences in socio-cultural factors and structures of healthcare systems.
2. The prevalence rates of victimization among people with SMI reveal striking gender differences compared to the general population. We aim to explore the significance of gender in experiences of violence within both heterosocial and homosocial contexts, drawing on socio-cultural and socio-structural gender theories that frame violence as an expression of status positions.
3. Since violence experiences among people with SMI often exhibit an overlap between victimization and perpetration of violence, we aim to gain insights beyond rigid attributions of (male) perpetrator status versus (female) victim status, and we shed light on the forms and mechanisms of perpetrator-victim-overlaps. This approach seeks to undermine a dichotomous understanding of people with SMI as either victims or perpetrators.
4. Quantitative studies provide limited insights into the complexity of violence experiences. Due to the temporal structure of violence experiences, we investigate experiences of violence through a biographical approach, which appears particularly suitable for investigating serial or re-victimization as well as biographical continuities, disruptions, and transformations in the violence experiences.
5. There are only a few studies on the perceptions, considerations, and the therapeutic management of mental health service users who experienced violence, as well as on the support needs of psychiatric professionals in dealing with their clients’ violence experiences. By investigating perspectives of mental health professionals in dealing with service users’ experienced violence and their disclosure, we aim to gain insights into how mental health professionals’ attitudes impact the opportunities for disclosing violence experiences in psychiatric settings.

Our study aims to contribute fundamentally to a deeper understanding of violence experienced by people with SMI in Germany, thereby providing the groundwork for the development of targeted interventions to improve awareness and the response to such experiences within this particularly vulnerable group.
