# Correction: Experiences of violence among people with severe mental illness: social, biographical and institutional factors (EVIO). A study protocol for a mixed-methods study

**DOI:** 10.3389/fpsyt.2026.1862825

**Published:** 2026-05-12

**Authors:** Silvia Krumm, Anita Scheuermann, Claudia Helmert, Paula Kittelmann, Melanie Pouwels, Nicolai Hojka, Jana Karp, Georg Schomerus

**Affiliations:** 1 Department of Psychiatry and Psychotherapy II, Ulm University, Ulm, Germany; 2 Department of Psychiatry and Psychotherapy, University of Leipzig, Leipzig, Germany; 3 ”Vorurteilsfrei” registered nonprofit association, Leipzig in cooperation with the Universities of Ulm and Leipzig, Leipzig, Germany

**Keywords:** mental health professionals, mental health service users, mental illness, mixed-methods study, prevalence rate, qualitative study, victimization, violence

The funder Leipzig University “(Open Access Publishing Fund)” was not included in the original version, as confirmation of **Funding** was received after the author proof stage.

The correct **Funding** statement reads:

“The research is funded by the German Research Foundation (Deutsche Forschungsgemeinschaft) under project number 510625985. Supported by the Open Access Publishing Fund of Leipzig University. The funders were not involved in the design of the study, data collection and analysis or writing of the manuscript.”

The original version of this article has been updated.

